# Auditory event-related potentials based on name stimuli: A pilot study

**DOI:** 10.3389/fnins.2022.808897

**Published:** 2022-09-01

**Authors:** Jindi Zhao, Yuancheng Yang, Xingwei An, Shuang Liu, Hongyin Du, Dong Ming

**Affiliations:** ^1^Academy of Medical Engineering and Translational Medicine, Tianjin University, Tianjin, China; ^2^College of Precision Instruments & Optoelectronics Engineering, Tianjin University, Tianjin, China; ^3^Department of Anesthesiology, Tianjin First Central Hospital, Tianjin, China

**Keywords:** auditory event-related potentials, name stimulation, classification and recognition, P300, P200

## Abstract

In recent years, diagnostic studies of brain disorders based on auditory event-related potentials (AERP) have become a hot topic. Research showed that AERP might help to detect patient consciousness, especially using the subjects' own name (SON). In this study, we conducted a preliminary analysis of the brain response to Chinese name stimuli. Twelve subjects participated in this study. SONs were used as target stimuli for each trial. The names used for non-target stimuli were divided into three Chinese character names condition (3CC) and two Chinese characters names condition (2CC). Thus, each subject was required to be in active (silent counting) and passive mode (without counting) with four conditions [(passive, active) × (3CC, 2CC)]. We analyzed the spatio-temporal features for each condition, and we used SVM for target vs. non-target classification. The results showed that the passive mode under 3CC conditions showed a similar brain response to the active mode, and when 3CC was used as a non-target stimulus, the brain response induced by the target stimulus would have a better interaction than 2CC. We believe that the passive mode 3CC may be a good paradigm to replace the active mode which might need more attention from subjects. The results of this study can provide certain guidelines for the selection and optimization of the paradigm of auditory event-related potentials based on name stimulation.

## Introduction

Disorder of consciousness (DOC) caused by severe brain injuries includes minimally conscious state (MCS) and vegetative state/unresponsive wakefulness syndrome (VS/UWS) (Giacino et al., 2014). MCS presents a small number of perceptual behaviors, while VS presents a basic loss of understanding about itself and the environment (Kotchoubey et al., 2005; Laureys et al., 2010). The research on MCS and VS has important significance in both the clinical level and scientific field. Accurate diagnosis and effective wake-up treatments of these two brain states are one of the most urgent problems that need to be solved (Gosseries et al., 2011). At present, various rating scales are commonly used in the clinical evaluation of DOC patients, while the rate of misdiagnosis is up to 40% since the rating method depends much on patients' behavior ability and is subjectively affected by doctors. To reduce misdiagnosis and improve the quality of prognosis, many other methods have been used to assess consciousness (Holeckova et al., 2006; Di et al., 2007; Blankertz et al., 2011; De Martino et al., 2011; Phillips et al., 2011; Jox et al., 2012; Kurz et al., 2018).

Auditory event-related potential (AERP) is a non-invasive electrophysiological test with millisecond resolution that can distinguish the relationship between cognitive electrophysiological components and different cognitive stages (Polich et al., 1990). It has been increasingly used in the diagnosis and evaluation of clinical cognitive impairment (Golob et al., 2002; Howe et al., 2014; Morrison et al., 2018). AERP reflects the entire process from cochlear activation to higher cognitive processing, providing objective and neurophysiological information about the brain's response to auditory stimuli (Cowan et al., 1993). In recent years, auditory stimuli with emotional characteristics have been increasingly used by researchers, such as preferred music, animal sounds, natural sounds, names, and so on (Heine et al., 2015; Perrin et al., 2015; Wu et al., 2018; Carriere et al., 2020). Names are the words that people are most familiar with, and many researchers have revealed the self-priority of names (Nakane et al., 2015; Kotchoubey and Pavlov, 2017; Blume et al., 2018). As far as clinical arousal therapy is concerned, the use of name-auditory evoked potentials (NAEP) is one of the commonly used clinical methods (Thul et al., 2016; Onishi et al., 2017; Crivelli et al., 2020). Due to the particularity of names, many researchers have begun to use SON to assess the state of consciousness in patients with brain injury (Holeckova et al., 2008; Real et al., 2016). Kempny et al. (2018) recruited 12 healthy subjects and 16 DOC patients for EEG experiments, and they randomly inserted SON into a sequence composed of others' names (ONs), extracted ERP signals, and used statistics parameter mapping (SPM) for analysis. The results showed that there were no significant differences in SON and ON between 12 patients at the group level, and only 4 patients had similar ERP responses to the healthy group, with significant differences in ERP under different name stimuli, which demonstrated the feasibility of using name stimuli to assess DOC patients. According to EEG studies, SON can induce ERP and beta power inhibitory components (Tamura et al., 2016) and can be used to improve the prognostic value of ERPs in coma patients and to evaluate the cognitive process of unresponsive patients (Fischer et al., 2008; Giacino et al., 2018). The study of Tacikowski and Nowicka (2010) also showed that the response time of one's own name to stimuli was shorter than others' names, and the induced P300 amplitude was stronger. The “calling a person's name” method has been included in the latest guidelines for consciousness disorders in the United States, which shows that compared with meaningless sounds, individual-related name stimulation is more effective and provides a better cognitive state (Bekinschtein et al., 2004), and it is a stimulus that is conducive to promoting cognition because name stimuli are more activated in the temporal lobe (Wu et al., 2018).

At present, many studies (Naci and Owen, 2013; Zhu et al., 2019) have not only verified the cognitive neural mechanism of name stimulation but also applied it to the clinical field, providing good help for the consciousness detection and diagnosis of brain injury patients. As a kind of sensory stimulation, the auditory mode can effectively provide a good cognitive environment by calling the patient's name, which can effectively activate the relevant brain regions, and the difference between different levels of consciousness can be seen by fMRI or EEG technology (Qin et al., 2008; Cheng et al., 2013). Calling patients' names has become an important topic in the field of consciousness diagnosis internationally and has been widely used in clinical practice with significant advantages.

However, there are still some remaining problems in current research: First, existing research on the mechanism of interaction is mainly developed from the perspective of active attention, but there are few reports on whether there is a cognitive integration process under non-attentional conditions. Second, previous studies on ERP have reported enhancement of own name response relative to “non-target” names such as strangers, celebrities, newly familiarized names, and so on. Those studies focus on the difference of different familiarity of the names, while few studies reported the brain response to the characteristic of the “non-target” names. Does this particular reaction interact when the subject responds to the target stimulus? In China, most people have a name with Three Chinese Characters which could be noted as three syllables (3CC, such as /li/ /xiao/ /long/ and /liu/ /da/ /si/) or Two Chinese Characters which could be noted as two syllables (2CC, such as /san/ /zhang/ and /wang/ /si/). What are the differences between two-character and three-character names? Why would it differentially impact own name recognition in a meaningful way? There is no relevant research to prove the above view so far.

In this study, subjects were required to be in both active mode (counting silently) and passive mode (only listening) with P300 induced by SON as the target stimulus and other names (ONs) as the non-target stimulus. We verify the brain response by studying the temporal and spatial information of EEG signals. In addition, to compare the target name detection ability from non-target stimuli, we compared the classification accuracies of different conditions based on the SVM method, which is usually used in pattern classification.

## Materials and methods

### Participants

Twelve healthy volunteers (6 males and 6 females; mean: 22.7 ± 0.75 years old) were tested in the experiment, all of them were college students. The subjects were all right-handed, reported no psychiatric disorders, had normal vision and hearing, none suffered from brain damage or had taken psychotropic drugs, did not drink coffee, tea, alcohol, or other beverages for 24 h before the experiment, and ensured adequate rest the night before the experiment according to subjects' oral report. All participants' first and immersed language was Chinese, and informed consent was obtained after understanding the process and purpose of this experiment by every subject. Of the 12 subjects, 6 people's names had Two Chinese Characters which could be noted as two syllables and the remaining 6 people had Three Chinese Characters which have three syllables.

### Stimuli and experimental procedure

In this study, the sound stimulus was recorded by a native Chinese male using a MOTU828ES sound card and an MXL-67i microphone. The recorder read more than 30 names on the list, including the subject's name, and each name was read aloud twice. The test audio was adjusted to the subject's comfortable sound intensity of 22 ±3 dB, with a sampling rate of 48 kHz, sampling accuracy of 24bit, and dual-channel acquisition. All sound files were edited by Adobe Audition software with the same stimulation duration and inter stimuli interval.

Due to the different types of Chinese names, the name stimuli were divided into Three Chinese Character names (3CC, such as /li/ /xiao/ /long/ and /liu/ /da/ /si/) and Two Chinese Character names (2CC, such as /san/ /zhang/ and /wang/ /si/). The sequence included five names with the SON as the target stimulus and four ONs as the non-target stimulus. The non-target stimulation was chosen for names that the subjects did not know, and the subjects were asked if they were familiar with the name before conducting the pre-experiment. All names before the experiment have been adjusted to replace and remove names that may produce acoustic similarities.

**Figure 1:**
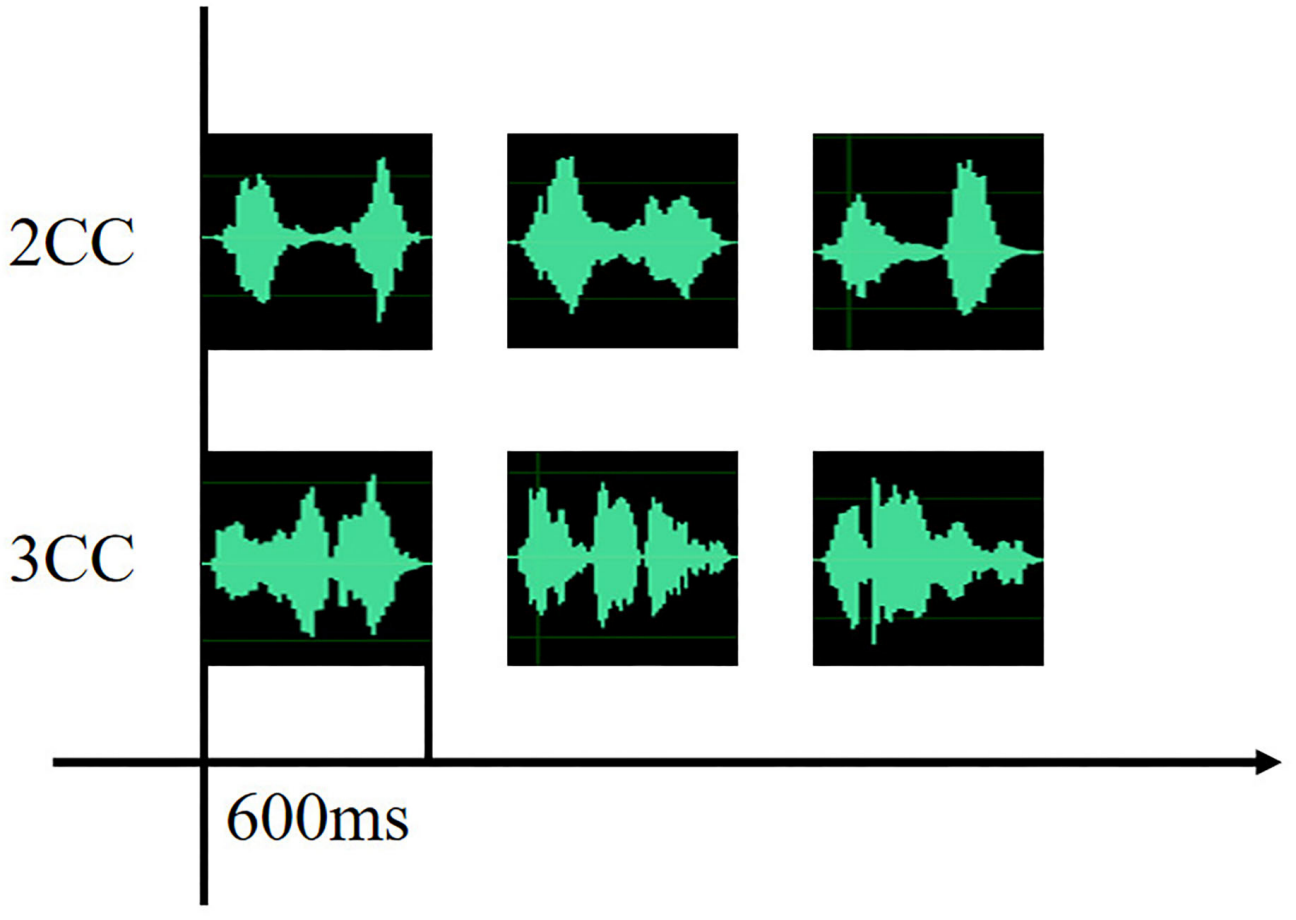
Sound signal waveforms of 2CC and 3CC.

Stimuli were arranged repeatedly in a pseudo-random order, which means that the two adjacent stimuli could not be the same. Before the experiment, the duration of each name was set to 600 ms using professional audio clip software (Adobe Audition CS6), and the inter stimuli interval (ISI) between every two adjacent auditory stimuli was randomly distributed between 500 and 800 ms transmitting by headphones. The sound signal waveforms of 2CC and 3CC are shown in Figure 1.

**Figure 2:**
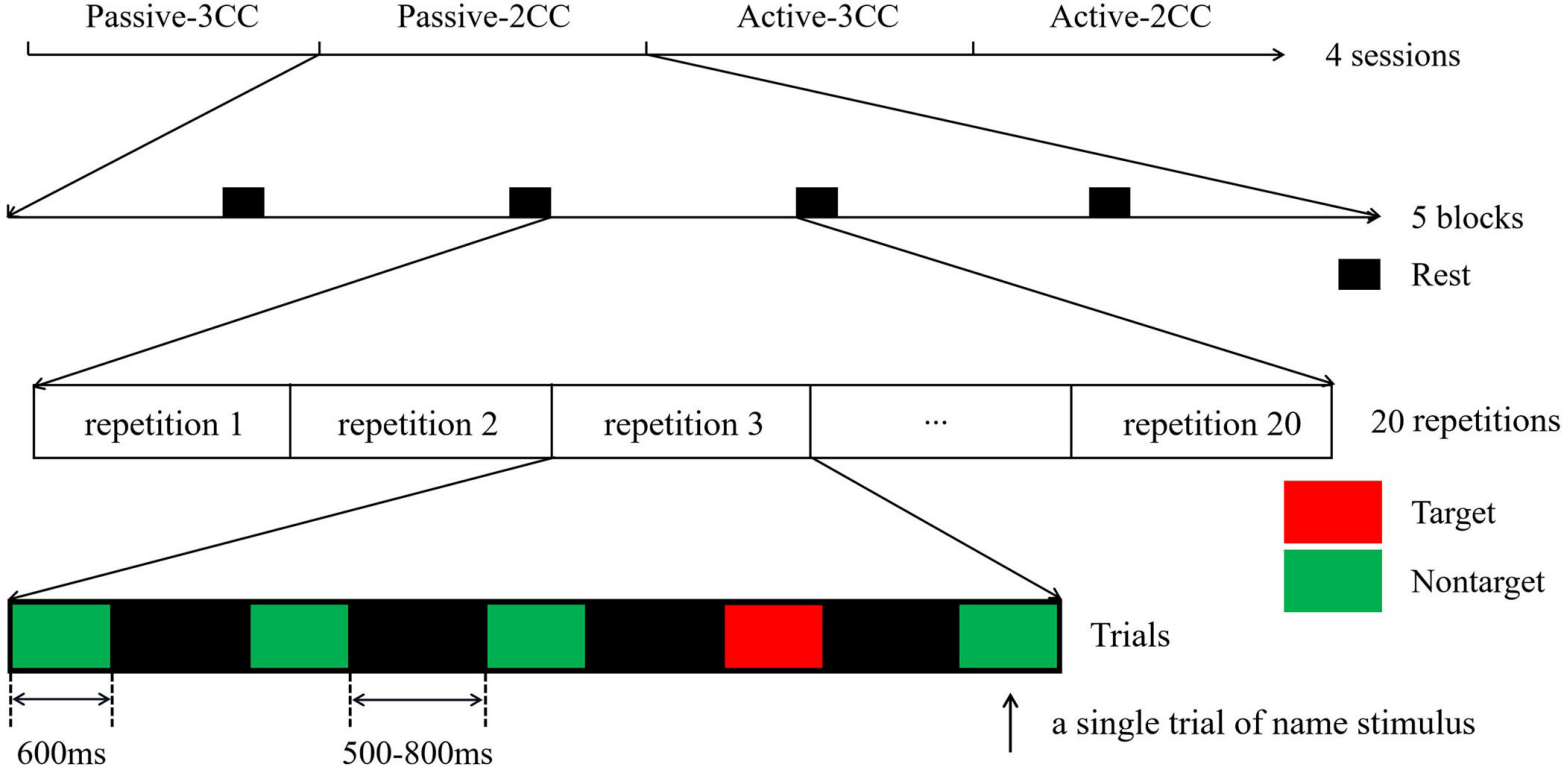
Experimental process chart.

The paradigm was designed by E-prime 3.0 system. Each experiment included five names, and these five names must appear in each repetition with a random presentation. In addition, it was necessary to ensure that the same stimulus occurred discontinuously to avoid overlapping in the evoked response. There were five blocks in one session. For each block, there would be 20 repetitions, each repetition containing five name stimuli, and the length of the name stimuli was 600 ms. Figure 2 showed the flow of the entire experiment, with red squares as target stimuli (SON), green as non-target stimuli (ON), and black as ISI.

During the active task, subjects were asked to pay attention to the sound stimuli they heard and to mentally count the appearance of subjects' own names (SONs); and during the passive task, subjects were asked to simply listen quietly, with no other requirements.

Subjects sat in a comfortable chair ~0.7 m from a 24-in LED monitor and were repeatedly instructed to avoid blinking and body movement. Before the formal experiment, a group of sound exercises was conducted to familiarize the participants with the task requirements and the experimental process. Subjects were explained the difference between target stimulus, non-target stimuli, active task, and passive task before each session. The whole experiment was conducted in a soundproof room.

In this experiment, subjects were asked to be in passive (only listening) or active (counting in silence) states. Each subject performed four experimental sessions corresponding to non-target 3CC stimuli of passive condition (3CC-passive), non-target 2CC stimuli of passive condition (2CC-passive), non-target 3CC stimuli of active condition (3CC-active), and non-target 2CC stimuli of active condition (2CC-active) in a sequential order. The experimental process was shown in Figure 1. At the beginning of each block, there was a visual cue “If you are ready, please press the SPACE key” lasting for 2 s, with clicking the space bar as a starting condition. All participants underwent the same experimental procedure, for 100 trials in one session, with 500 samples in total.

### EEG pre-processing

The EEG data were pre-processed including filtering, down-sampled, and independent component analysis (ICA). The pre-processing was completed by the EEGlab toolbox of MATLAB. Unwanted components and features in the signal are first removed by filtering, and then the data size is reduced by downsampling to facilitate subsequent processing. Next, artifact removal is performed by ICA, which is an inverse process that decodes from the input signal layer by layer outward to restore each of the mixed signals and thus obtain the original signal. An FIR band-pass filter with 0.5–40 Hz was used for offline filtering and downsampled to 200 Hz. Eye movement artifacts and motion artifacts were removed according to ICA results. The EEG data from 200 ms before stimuli to 800 ms after was regarded as the data segment of this stimulation, called an epoch, and all epochs were divided into target and non-target groups according to the type of stimulation. Epochs were subtracted from the average amplitude of the baselines, which were calculated at intervals of −200 to 0 ms before the stimuli onset. Some epochs were excluded if the amplitude was detected to exceed ±100 μV.

### Feature extraction

High domain features might bring bad classification performance when the training samples are limited. Thus, we conducted a feature extraction (selection) method before classification.

For feature extraction, *r*^2^ is used as the basis for differentiability judgments (Chum et al., 2012). The formula is as follows:


(1) $$r^{2}\left(t\right)=\pm {\left\{\frac{\left(\sqrt{M_{T}M_{N}}(\text{mean}\left(X_{T}\left(t\right)\right)-\text{mean}(X_{N}(t))\right)}{\left(M_{T}+M_{N}\right)std\left(X_{T}(t)\cup X_{N}(t)\right)}\right\}}^{2}$$

where *t*, *t* = 1, 2, …, *N*, is the time point of features. *X*_*T*_ and *X*_*N*_ represent the target feature vector and non-target feature vector, respectively. *M*_*T*_ and *M*_*N*_ represent the number of target samples and non-target samples as well. *mean* is to calculate the average value and *std* is to calculate the value of standard deviation. The average value of target minus non-target determines the sign of *r*^2^.

### Feature selection method

Based on the *r*^2^ results, we conducted the feature selection based on the following steps:

1) According to the ERP components, two constraint time windows were set (T1: 100–300 ms; T2: 300–600 ms), since most of the significant *r*^2^ were limited in these two time windows.
2) Calculated the *r*^2^ *score* in the first window:
$$\begin{array}{l} score\left(t\right)=mean\left(r^{2}\left(t\right)\right)+\max\left(r^{2}\left(t\right)\right) \end{array}$$
3) Calculated the correlation of adjacent time points in turn:
$$\begin{array}{l} c\left(t,\Delta t\right)=\;\frac{<x\left(t\right),x\left(\Delta t\right)>}{\left|x\left(t\right){|}^{*}|x\left(\Delta t\right)\right|} \end{array}$$
  *c* represents the correlation between two vectors, ranging from 0 to 1. Correlation increases as *c* increases.

The time range (t_r_(Ti,i=1,2)_) was chosen to satisfy more than half of the score value and the *c* value >0.7. The amplitudes in the time range were averaged as a feature. Thus, we get the feature in this t_r_(Ti)_. f_(Ti)_ = mean(*X*_*t*_*r*(*Ti*)_*(t)*).

Calculated the next constraint window, and repeated steps 2 to 4.

Finally, two features f_(T1)_ and f_(T2)_ in the two constraint time windows of T1 and T2 were obtained. For each subject, we have 500 samples (100 targets and 400 non-targets) and for each sample, we have 2 features ^*^ 60 channels. Thus, for each subject and each condition, a 500 ^*^ 120 matrix was obtained. The feature vectors corresponding to the target and non-target trials were labeled +1 and −1, respectively.

### Classification

The classification rate between targets and non-targets in each condition was calculated by a support vector machine (SVM) classifier. SVM is a generalized linear classifier, which transforms the actual problem into a high-dimensional feature space through a non-linear transformation, and constructs a linear discriminant function in the high-dimensional space to realize the non-linear discriminant function in the original space. Take binary classification as an example, the algorithm separates these two classes by dividing the hyperplane in the feature space of samples, and the two heterogeneous samples closest to the hyperplane are used as support vectors, and the distance between the two samples is called “interval.” The basic idea is to construct the optimal hyperplane in the feature space so that the distance between the hyperplane and the set of samples of different classes is maximized to achieve the maximum generalization ability (Bishop and Nasrabadi, 2006; Jordan and Mitchell, 2015).

SVM with a 10-fold cross-validation (Fushiki, 2011) method was used to perform the single trial classification accuracy, which means that it will classify each sample as target or non-target. We also performed the classification performance using 1 to 10 repetitions. For example, five repetitions mean that we will compare the classification decision values for each name in the first five trials for each block, and calculate the average decision value of each name. The name with the highest decision value will be classified as the target, otherwise the name will be classified as non-target. After that, receiver operating characteristic (ROC) and area under the roc curve (AUC) were used to obtain more evidence. Statistical analysis was to compare the different results among the conditions by *t*-test.

## Results

### Spatio-temporal characteristics of different conditions

**Figure 3:**
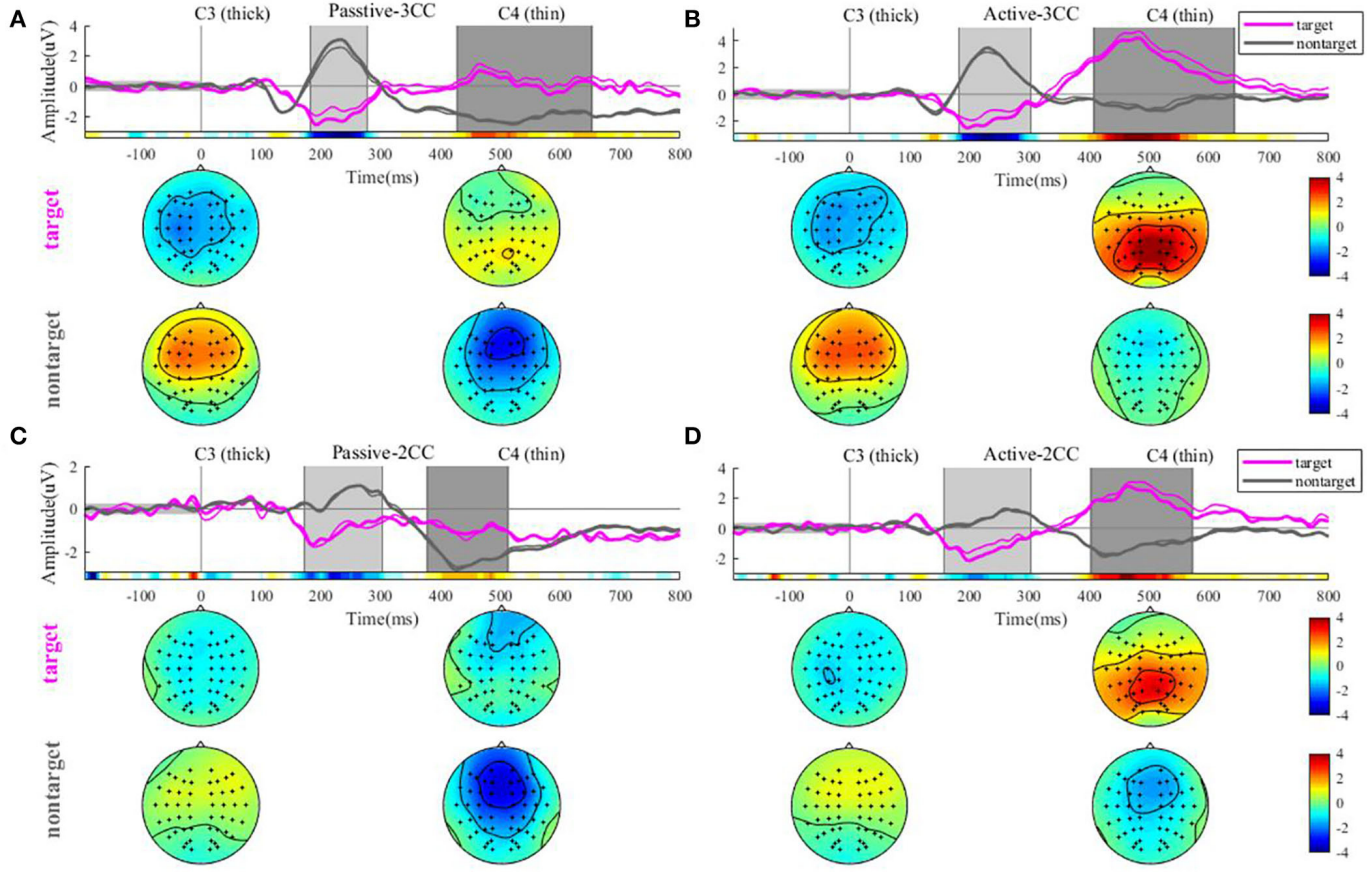
Average ERP waveforms at C3 and C4 electrodes of 12 people under 4 conditions in sequence. In each subfigure, **(A–D)** the pink and gray colors denote target and non-target stimuli, and the thick and thin lines denote C3 and C4 channels, respectively. The gray rectangle represents the time intervals that have a significant difference, and the colored long rectangle under the lines represents the sign *r*^2^ results for the entire time period. The circle below the waveform graph is the average amplitude topographic map in the first and second time intervals.

Figure 3 illustrates the AERP response and amplitude topographic map by an average of 12 people in 4 conditions. The four pictures represent passive-3CC, active-3CC, passive-2CC, and active-2CC, respectively. The pink line represents the results of target stimuli and the gray line represents the results of non-target stimuli. As shown in Figures 3A–D, the target stimuli in the active state could induce a marked P300 response at about 400 to 500 ms, while an obscure P300 can be observed only when the non-target was 3CC for the passive state.

The rectangular bar of *r*^2^ shows the area of two time windows that have a significant difference (or higher *r*^2^) marked with a gray rectangular window, respectively. By observing the brain map in figures A and B for results of 3CC conditions, it can be seen that those target stimuli induce a negative activation (might be noted as an N2 component) in 200 to 300 ms. Conversely, it evokes a high-energy activation (which might be noted as a P300 component) in the occipital region at 400 to 600 ms. However, non-target stimuli show the exact opposite of the target stimuli. The former showed activation of the central anterior gyrus and frontal regions in both areas, a positive reaction in the first gray time interval, and a negative reaction in the second gray time interval. This result can also be seen in figure C and figure D for results of 2CC conditions.

**Figure 4:**
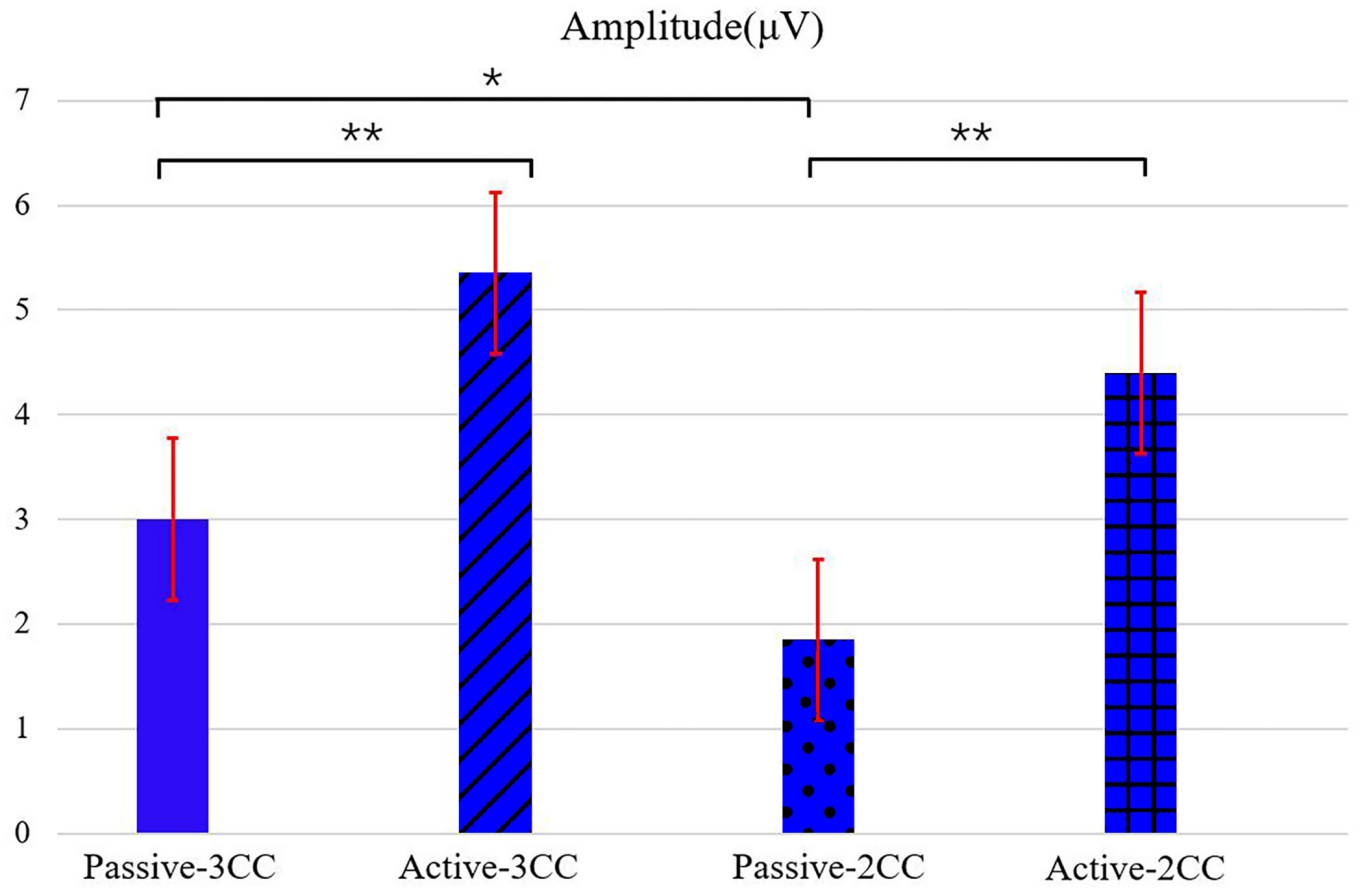
ERP amplitudes in the second time intervals (which indicated the P300 component) evoked by target stimuli in both passive and active conditions with different types of non-target stimuli. The a-black solid asterisk indicates a significant difference (*p* < 0.05) between two conditions, and the two-black solid asterisks indicate a very significant difference (*p* < 0.01) between two conditions. Values are the means and standard error (Mean ± SE) of P300 amplitude among an average of 12 people.

Figure 4 shows the ERP amplitudes in the second time intervals (see Figure 3, which indicated the P300 component) evoked by target stimuli. The means and standard error (Mean ± SE) of P300 amplitudes of 12 participants: passive-3CC (3.02 ± 0.76) μV, active-3CC (5.40 ± 0.72) μV, passive-2CC (1.87 ± 0.81) μV, and active-2CC (4.43 ± 0.73) μV. The amplitudes of P300 components in the active mode were significantly higher than that in passive mode, and the P300 amplitude induced by target stimuli in the active-3CC condition is the highest, while that in passive-2CC is the lowest. A significant difference in the ERP amplitude in the second gray time interval between passive-3CC and passive-2CC (*p* = 0.015) is found, which means the brain responds differently to different non-target stimuli. The significantly larger amplitude between passive-3CC and active-3CC (*p* = 0.006) and passive-2CC and active-2CC (*p* = 0.001) were found, and these indicate that when the non-target stimuli were the same, the ERP amplitude of the active task was greater than that of the passive task.

From the analysis of the signed *r*^2^, we could find that the most important components are in the two time intervals. Thus, we set two time intervals T1 (200–300 ms) and T2 (300–600 ms), and used the introduced feature selection method in section Method to extract useful features. The extracted features will be used to make the classification of targets and non-targets.

### Comparison of 2CC and 3CC names

The averaged ERP waveforms of subjects with 3CC names and 2CC names were also calculated, respectively, and the AERPs of the Pz channel induced by target stimuli and the AERPs of the Fz channel induced by non-target stimuli were presented.

**Figure 5:**
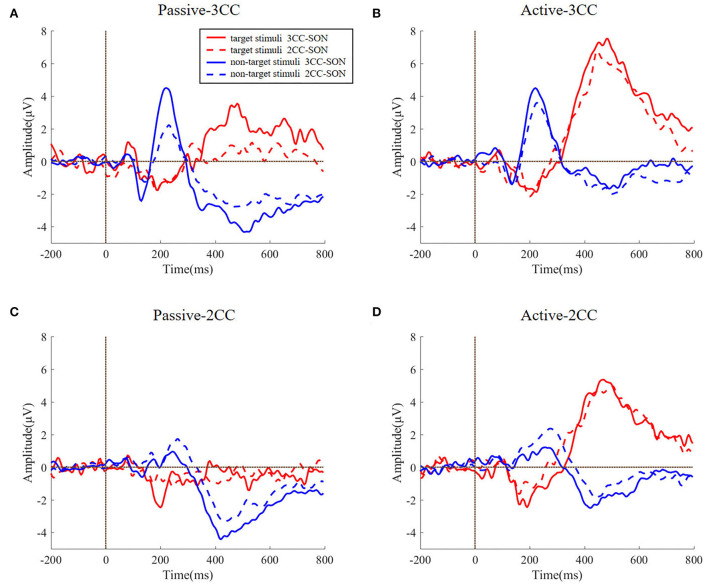
**(A–D)** The difference between the ERP evoked by the target stimulus and the non-target stimulus by the length of the subject's name. The four graphs, respectively, represent four task conditions. Lines of different colors represent different types of stimuli. The abscissa is time (ms) and the ordinate is amplitude (μV).

Figure 5 reflects the comparison of ERP waveforms under different types of subjects' names and different types of stimuli. Noticing that the AERPs of target stimuli were presented with the Pz channel and AERPs of non-target stimuli were presented with the Fz channels, we could find from Figure 3 that the target stimuli have strong response in parietal regions and the non-target stimuli have strong response at the frontal regions. The four sub-figures, respectively, represent passive 3CC condition, active 3CC condition, passive 2CC condition, and active 2CC condition. The red solid line and blue solid line, respectively, represent the ERPs of target and non-target stimuli when the subjects' own names are 3CC. The red dashed line and blue dashed line represent the ERPs when the subjects' names are 2CC. The horizontal axis is time and the range is 200 to 800 ms, and the vertical axis is the amplitude and the range is 5 to 8 μV.

It can be seen from Figure 5 that the target responses are similar no matter whether the SON names are 2CC and 3CC (*p* > 0.5), and the non-target stimuli are also similar (*p* > 0.5) even when the SON names are different (2CC or 3CC) in a specific condition. The *t*-test results could be found in Table 1. This proves that in this study, for the induced AERPs, the length of the subject's own name has no significant effect on the experimental results.

**Table 1:** The mean amplitudes and latencies as well as the *t*-test results.

**Targets Pz channel**	**SON** = **3CC**		**SON** = **2CC**		***T*** **-test (** ***p*** **-value)**	
	**Amplitude/μV**	**Latency/ms**	**Amplitude/μ*V***	**Latency/ms**	**Amplitude**	**Latency**
Passive 3CC	3.48	480	1.08	535	0.87	0.83
Active 3CC	7.46	485	6.69	445	0.83	0.27
Passive 2CC	0.36	395	1.20	450	0.33	0.82
Active 2CC	5.34	470	4.90	505	0.78	0.35
**Non-targets Fz channel**	**Amplitude/μ** *V*	**Latency/ms**	**Amplitude/μ** *V*	**Latency/ms**	**Amplitude**	**Latency**
Passive 3CC	4.48	220	2.18	230	0.99	0.47
Active 3CC	4.46	220	3.55	225	0.93	0.13
Passive 2CC	0.93	245	3.14	240	0.92	0.25
Active 2CC	1.21	255	2.35	275	0.89	0.10

### Classification and recognition

**Figure 6:**
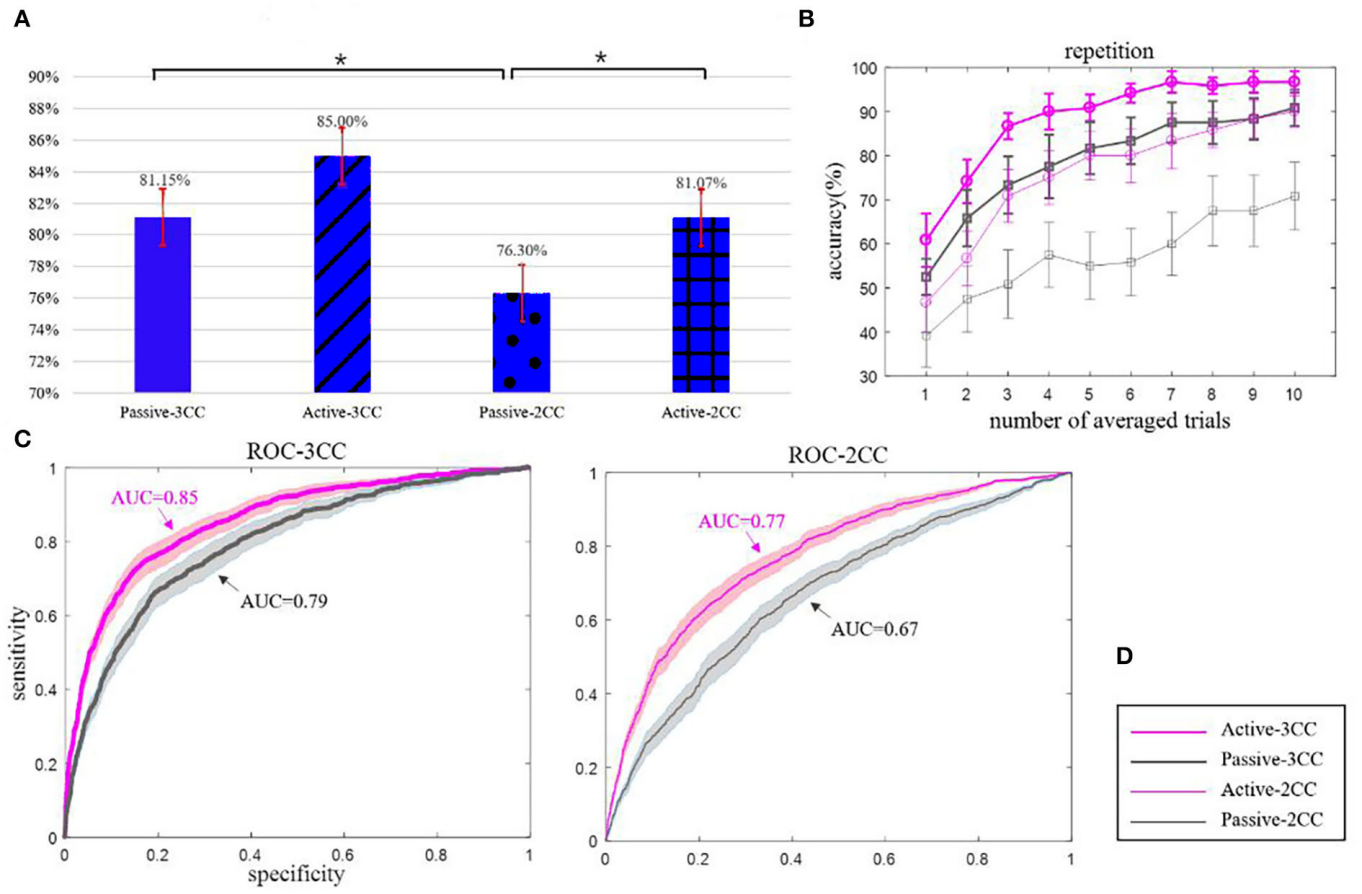
**(A)** represents the binary classification accuracy of four conditions, **(B)** represents the repetition results with the fold from 1 to 10, **(C)** represents the ROC curves of 3CC and 2CC non-targets, and **(D)** represents the legend. The thick and thin lines represent 3CC non-targets and 2CC non-targets, and pink and gray colors represent active and passive states, respectively. The black solid asterisks denote the significant differences. They are all average values of 12 people, and error bars and colored areas are the means and standard errors (Mean ± SE).

Figure 6 shows the classification of targets and non-targets using extracted features. Figure A and figure B indicate the binary classification and the averaged classification results of all subjects. Figure 6A shows the performance of binary classification; since the samples of the targets and non-targets are significantly unbalanced, we use the average of four times binary classification results as the final binary results. For each participant, we have one SON and four ONs, with each name having 100 samples. Thus, we classified the SON (100 samples) with each ON (100 samples) to get the binary classification. At last, we will average the performance of four binary classification as the final performance.

The means and standard error (Mean ± SE) of accuracy (Figure 5A): passive-3CC (81.15 ± 1.71) %, active-3CC (85.00 ± 1.74) %, passive-2CC (76.30 ± 1.63) %, and active-2CC (81.07 ± 1.69) %. The mean single-trial classification accuracy in active-3CC is 85% while that in passive-3CC is 81.15% with an insignificant difference (*p* = 0.086). The mean accuracy in active-2CC is 81.07% and in passive-2CC is 76.3% with a significant difference by *t*-test (*p* = 0.015). There is no significant difference between the accuracy rate of 3CC and 2CC under active conditions (*p* = 0.052), but the difference is significant under passive conditions (*p* = 0.027). In addition, passive-3CC (81.15%) and active-2CC (81.07%) showed similar classification accuracy.

Figure 6B shows the performance of the name classification performance with different repetitions of stimuli, each repetition has five trials (names). We use the classifier to make a binary classification of each trial providing the decision value for each class. After a repetition (or repetitions), the decision values for each name as the target stimuli were calculated and compared, and the name with the highest decision value would be classified as the target stimuli.

Figure 6B shows that the accuracy of 10 repetitions could exceed 90% under three conditions, while that is only 70% in the passive-2CC condition. According to the statistical analysis, there is no significant difference in accuracy rate between active and passive in the case of 3CC non-target (*p* = 0.1), and the difference in the case of 2CC non-target is very significant (*p* = 0.003). There is no significant difference between passive-3CC and active-3CC, and the accuracies of passive-3CC and active-2CC were relatively close in each average trial, which reveals the possibility of passive mode replacing active mode and provides evidence for the feasibility of the experimental scheme in this study.

As can be seen from Figure 6C, the AUC of active-3CC is larger than that of passive-3CC, the AUC of active-2CC is also larger than that of passive-2CC, and the curves of active-3CC and passive-3CC are better than that of active-2CC and passive-2CC. In addition, when the non-target is 3CC, the ROC curve effect and the AUC value obtained are both greater than those of the 2CC non-target. Besides, under the 3CC condition (*p* = 0.13), there is no significant difference between the active and passive results, while the difference is very significant under the 2CC condition (*p* = 0.012). This result reveals that the effect of the passive-3CC classifier is close to that of active-3CC and active-2CC classifiers, which verifies the above conclusion.

## Discussion

In the brain, each stimulus will go through a series of complex procedures including language perception, cognitive integration, and decision processing, involving the cooperation of different brain regions (Bressler and Menon, 2010), and is affected by the attention and familiarity of individuals. This study uses quantitative EEG analysis to investigate whether different types of stimuli can cause changes in brain responses when the participants' attention changes. In this study, the non-target stimuli of 3CC were able to play a facilitating role in the brain's recognition of target stimuli, producing higher amplitudes and better correct classification rates than the non-target stimuli of 2CC, providing a theoretical direction for studying the interaction between target and non-target stimuli in the auditory paradigm; in addition, the passive 3CC task paradigm used in the present study evoked a brain response which was similar to that of the active task, and although it produced a lower P300 amplitude, it showed similar classification results in classification recognition as the active 2CC task, revealing the feasibility of using a 3CC name design auditory paradigm to detect the level of consciousness in a passive auditory task based on Chinese names.

### Non-target stimuli play a key role in the brain's cognitive processes

In this study, subjects were required to be in a passive listening state and active counting state to research the P300 potential (the positive component of about 500 ms in the T2 time zone) by oddball paradigm, by classifying the non-target stimuli into different types to determine whether the P300 responses were similar in the four tasks. Notably, the non-target stimuli were the same for all subjects under the same conditions, and the only change was caused by the target stimulus. This was expected in this study because the experiment required the subjects to perform mental counting of the target stimulus in the active task. At this time, the brain could produce special neural activity to the stimulus, thus inducing a strong P300. Because in the passive state, an obscure P300 can only be observed when the non-target is 3CC; therefore, we think that when the non-target stimulus is 3CC, it would affect the brain's response to the target stimulus, resulting in a large ERP response. Moreover, from Figure 3, the brain response differs for different non-target stimuli, and the magnitude of 3CC is >2CC in both active and passive states, which reflects the dominance of long names. Therefore, we believe that the brain response to non-target stimuli will be influenced by the type of non-target.

Various psychological studies have proved that strong attention can be led by hearing one's own name which is related to personal emotions (Snyder et al., 2012), and the auditory threshold for perceiving one's own name is lower than other names (Howarth and Ellis, 1961). To some extent, MCS and even VS/UWS patients can distinguish their own names and other names (Fellinger et al., 2011). Schnakers (Schnakers et al., 2008) showed that the amplitude of P300 in MCS patients is different in active and passive modes; Cavinato (Cavinato et al., 2009) held that P300 is an indicator that can predict the recovery of consciousness in VS patients by using SON as deviant stimulation. The results of this paper show that the brain response is different for different non-target stimuli, and for subjects, whether the non-target stimulus is 2CC or 3CC, the subject's own name does not change (the target stimulus remains the same), and the brain's response to the stimulus evoked by the subject's name should be theoretically the same, but the results of this paper show that if the brain's response to the non-target stimulus 2CC and 3CC is different, then we believe that the different non-target stimuli affect the brain's response to the subjects' own names. In addition, because the stimuli in this study were rapid stimuli, when 2CC and 3CC were used as non-target stimuli to the brain, the responses were still in the process of continuation, which could affect the brain's response to the target stimuli. Therefore, it is important to study the brain responses evoked by different types of non-target stimuli.

### The passive mode may be an alternative paradigm to replace the active mode

From the results in Figure 6, it shows that there is no significant difference between passive-3CC and active-3CC (*p* = 0.1 in repetition and *p* = 0.13 in ROC), which proves that the brain response under the two conditions is similar, that is, the brain's ability to recognize target stimuli is similar. To a certain extent, this provides evidence for using a passive auditory paradigm instead of an active auditory paradigm for DOC patients. Previous studies have shown that stronger self-related information induces larger ERP responses, such as P200 and P300, both during the conscious period (Fan et al., 2013; Tacikowski et al., 2014) and coma state (Lancioni et al., 2010). Therefore, the auditory paradigm, especially the name stimulus, has been widely used in clinical research, providing an auxiliary role for the conscious diagnosis of DOC patients (Fischer et al., 1999; Di et al., 2007).

The “self-name effect” indicates that the self-processing related to names has a very responsible mechanism, which may include two different stages of early and late processing including P200 (positive components around 200 ms) and P300 (Perrin et al., 1999). Repeated training of auditory can generate ERP waveforms (Baykara et al., 2016), and the amplitude of P300 reaches its maximum value in the parietal lobe (Key et al., 2016). The P200 component is an early factor reflecting the process of perception and one of the objective indicators reflecting cognitive dysfunction. It is specifically related to the evaluation of attention and memory function. But there is only a P300 can be observed without a P200 response induced by target stimuli in our experiment. On the contrary, non-target stimuli induce an early response of the P200 response. To our surprise, the brain's response to the target stimuli is also affected by the types of non-target stimuli. Both in a passive or active condition, 3CC of non-targets has a clear advantage over 2CC of non-targets, such as higher P300 amplitude and better classification accuracy.

In summary, the purpose of this study was to verify the rationality of using name simulation as self-relevant information in healthy subjects during a monotonous auditory task based on the oddball paradigm. The analysis indicated that the P300 amplitude was higher in active conditions than that in passive conditions, and the P200 could be captured evoked by non-targets with no difference between the active and passive conditions. Moreover, when the P200 and P300 features were used as input to the SVM, the single-trial accuracy rate reached 80%, and the trial-averaged accuracy exceeded 90% in all conditions except passive-2CC. Our findings, although limited by the small sample size and confined reliability, suggest the possibility of a detection model and analysis method based on the passive paradigm. We expect to replace the active response paradigm by designing a passive auditory ERP paradigm, which requires a larger sample to focus on the relationship between auditory stimuli and consciousness. Future research needs to further study the ERP paradigm of unresponsive crowds, establish a standardized database, and use this approach to assess the residual self-consciousness of patients.

### Limitations of the study

This study designed an AERP experiment based on name stimuli, and although we obtained relatively satisfactory results, there are still many shortcomings that can be further improved. First, this paper studied the brain responses produced by 12 subjects with limited names and limited stimuli evoked, and the results still need further improvement and validation; second, the time to complete a task in this paper is about 12 min, which is too long for clinical patients and may lead to subjects' fatigue and poor experience during the experiment. Optimization of the auditory paradigm to obtain better results in a shorter time is the main direction of future research; finally, this study takes the detection of the clinical level of consciousness as the background and tries to explore the passive auditory paradigm applicable to clinical use, but due to the impact of the COVID-19 epidemic, which makes this study no clinical patient experiments at present, but only data analysis based on healthy people and future when the situation improves. After the situation improves, the direction must be put into the clinic.

## Conclusion

The results showed that the brain response in the passive 3CC mode was similar to that in the active mode. When 3CC was used as a non-target stimulus, the brain response induced by the target stimulus had better interaction than that induced by 2CC. We believe that the brain response to non-target stimulation will be affected by non-target types, and passive mode 3CC may be a good example of an alternative to active mode. The results of this study can provide some guidance for the selection and optimization of the name-based auditory event-related potential paradigm.

## Data availability statement

The raw data supporting the conclusions of this article will be made available by the authors, without undue reservation.

## Ethics statement

The studies involving human participants were reviewed and approved by Tianjin University. The patients/participants provided their written informed consent to participate in this study.

## Author contributions

All authors listed have made a substantial, direct, and intellectual contribution to the work and approved it for publication.

## Funding

This work was supported in part by the Nation Key Research and Development Program of China (No. 2017YFB1300302) and the National Natural Science Foundation China (Nos. 81630051 and 61603269).

## Conflict of interest

The authors declare that the research was conducted in the absence of any commercial or financial relationships that could be construed as a potential conflict of interest.

## Publisher's note

All claims expressed in this article are solely those of the authors and do not necessarily represent those of their affiliated organizations, or those of the publisher, the editors and the reviewers. Any product that may be evaluated in this article, or claim that may be made by its manufacturer, is not guaranteed or endorsed by the publisher.
